# Supplementary material for: Genetic insights into family group co-occurrence in Cryptocercus punctulatus, a sub-social woodroach from the southern Appalachian Mountains
Source: PeerJ. 2017 Mar 23;5:e3127. doi: 10.7717/peerj.3127 (PMC5366060; doi:10.7717/peerj.3127)
Supplement: Appendix S1 [file peerj-05-3127-s001.docx]

**Garrick RC (2017) Genetic insights into family group co-occurrence in *Cryptocercus punctulatus*, a sub-social woodroach from the southern Appalachian Mountains. *PeerJ*.**

**Appendix 1**. Geographic locations of rotting logs from which adult *C. punctulatus* woodroaches were sampled. Abbreviations associated with region names are: Mountain(s), Mtn; National Forest, NF; National Historical Park, NHP; National Military Park, NMP; National Park, NP; Nature Preserve, Nat. Pres.; Parkway, Pkwy; State Park, SP; and Wildlife Management Area, WMA. Spatial coordinates are reported in decimal degrees, and elevation is in meters. Classes of rotting logs, assigned using individual-based information on mtDNA haplotype and sex, are: single-haplotype (SH) logs, multi-family (MF) logs, and other multi-haplotype (OMH) logs.

| **State** | **Region** | **Site name** | **Latitude** | **Longitude** | **Elevation** | **Number of individuals** | **Log classification** |
| --- | --- | --- | --- | --- | --- | --- | --- |
| Alabama | Bankhead NF | A131 | 34.41979 | -87.33273 | 321 | 3 | SH |
|  |  | A129 | 34.23058 | -87.36352 | 273 | 3 | OMH |
|  |  | A133 | 34.17659 | -87.27680 | 248 | 2 | OMH |
|  |  | A132 | 34.11238 | -87.22862 | 179 | 2 | MF |
|  |  | A128 | 34.06701 | -87.32520 | 249 | 3 | SH |
|  | Little River Canyon Nat. Pres. | A134 | 34.45540 | -85.58357 | 395 | 3 | SH |
|  | Shinbone Ridge | A138 | 34.14676 | -85.84679 | 188 | 3 | SH |
|  | Talladega NF | A137 | 33.96340 | -85.45730 | 300 | 3 | SH |
|  |  | A41 | 33.91858 | -85.49764 | 257 | 3 | SH |
|  |  | A120 | 33.70745 | -85.59404 | 360 | 3 | SH |
|  |  | A122 | 33.67281 | -85.62832 | 427 | 3 | MF |
|  |  | A119 | 33.57288 | -85.69289 | 340 | 3 | MF |
|  |  | A118 | 33.49199 | -85.77732 | 413 | 3 | OMH |
|  |  | A117 | 33.47105 | -85.80658 | 621 | 3 | OMH |
|  |  | A127 | 33.33344 | -86.02572 | 313 | 2 | OMH |
|  |  | A16 | 33.20150 | -86.07201 | 291 | 3 | MF |
|  |  | A116 | 33.20099 | -86.07185 | 301 | 3 | OMH |
|  |  | A136 | 34.35716 | -85.67106 | 392 | 2 | SH |
|  | De Soto Falls | A135 | 34.55167 | -85.59611 | 526 | 3 | SH |
| Georgia | Chattahoochee NF | A19 | 34.93135 | -84.65486 | 485 | 3 | MF |
|  |  | A27 | 34.91647 | -83.41197 | 871 | 3 | OMH |
|  |  | A18 | 34.87866 | -84.71137 | 354 | 3 | SH |
|  |  | A20 | 34.87367 | -84.56690 | 1033 | 3 | MF |
|  |  | A21 | 34.77507 | -84.33880 | 730 | 2 | OMH |
|  |  | A40 | 34.75931 | -84.69140 | 804 | 3 | SH |
|  |  | A17 | 34.75830 | -84.73613 | 353 | 3 | SH |
|  |  | A25 | 34.74192 | -83.73265 | 766 | 3 | MF |
|  |  | A22 | 34.68311 | -84.25093 | 810 | 3 | SH |
|  |  | A23 | 34.63553 | -84.17392 | 966 | 3 | SH |
|  | Cloudland Canyon SP | A85 | 34.84695 | -85.49971 | 315 | 3 | MF |
|  | Johns Mtn WMA | A06 | 34.64336 | -85.21630 | 386 | 3 | MF |
|  |  | A08 | 34.61649 | -85.22780 | 366 | 2 | SH |
|  |  | A09 | 34.56515 | -85.24268 | 408 | 3 | SH |
|  |  | A10 | 34.56416 | -85.24043 | 427 | 2 | SH |
|  |  | A11 | 34.54107 | -85.25067 | 341 | 3 | SH |
|  |  | A05 | 34.47900 | -85.16069 | 404 | 2 | SH |
| Tennessee | North Cherokee NF | A49 | 36.32630 | -82.11465 | 688 | 2 | SH |
|  |  | A44 | 36.31100 | -82.07211 | 648 | 2 | SH |
|  |  | A46 | 36.22184 | -82.00381 | 1121 | 3 | SH |
|  |  | A47 | 36.17875 | -82.10726 | 1026 | 3 | SH |
|  |  | A48 | 36.15363 | -82.25122 | 886 | 3 | OMH |
|  | Clear Creek | A75 | 36.12452 | -84.74478 | 378 | 2 | OMH |
|  | Great Smoky Mtn NP | A37 | 35.77140 | -83.21343 | 575 | 2 | SH |
|  |  | A31 | 35.70232 | -83.35717 | 653 | 3 | SH |
|  |  | A38 | 35.61933 | -83.66993 | 593 | 2 | OMH |
|  | South Cherokee NF | A42 | 35.10896 | -84.62477 | 530 | 2 | SH |
| North Carolina | Great Smoky Mtn NP | A36 | 35.71242 | -83.09622 | 1059 | 3 | OMH |
|  |  | A35 | 35.65682 | -83.07098 | 843 | 3 | SH |
|  |  | A33 | 35.61787 | -83.21023 | 953 | 2 | OMH |
|  |  | A28 | 35.60975 | -83.44732 | 1613 | 3 | OMH |
|  |  | A34 | 35.53114 | -83.17092 | 1421 | 3 | SH |
|  |  | A32 | 35.52117 | -83.31077 | 666 | 3 | MF |
|  | Pisgah NF | A98 | 35.74876 | -82.33347 | 1631 | 3 | MF |
|  |  | A96 | 35.47536 | -82.61497 | 916 | 3 | MF |
|  |  | A95 | 35.37037 | -82.79151 | 1387 | 3 | SH |
|  | Blue Ridge Pkwy | A97 | 35.59535 | -82.48742 | 722 | 3 | MF |
|  |  | A93 | 35.48555 | -83.15284 | 1449 | 3 | OMH |
|  |  | A94 | 35.32512 | -82.96377 | 1714 | 3 | OMH |
|  | Nantahala NF | A92 | 35.32969 | -83.59187 | 593 | 2 | SH |
|  |  | A90 | 35.27122 | -83.68566 | 683 | 3 | OMH |
| South Carolina | Sumter NF | A142 | 35.01376 | -83.05563 | 887 | 3 | OMH |
|  |  | A143 | 34.94523 | -83.08929 | 744 | 3 | MF |
|  |  | A46 | 34.77755 | -83.31242 | 469 | 3 | MF |
| Virginia | Shenandoah NP | A61 | 38.87407 | -78.20597 | 597 | 3 | SH |
|  |  | A62 | 38.80508 | -78.18149 | 755 | 3 | SH |
|  |  | A63 | 38.76103 | -78.28269 | 1051 | 3 | MF |
|  |  | A65 | 38.55696 | -78.37899 | 1047 | 3 | SH |
|  |  | A59 | 38.37977 | -78.51651 | 876 | 3 | MF |
|  |  | A58 | 38.22345 | -78.73357 | 894 | 3 | SH |
|  |  | A57 | 38.17707 | -78.76551 | 835 | 3 | OMH |
|  | Washington NF | A66 | 38.32792 | -79.27844 | 733 | 3 | SH |
|  |  | A67 | 38.22689 | -79.41093 | 657 | 3 | MF |
|  |  | A69 | 38.11541 | -79.34189 | 573 | 2 | SH |
|  |  | A68 | 38.07403 | -79.44785 | 669 | 3 | SH |
|  | Jefferson NF | A54 | 37.46684 | -79.69961 | 651 | 3 | SH |
|  |  | A51 | 36.73672 | -81.43256 | 874 | 3 | SH |
|  |  | A50 | 36.64575 | -81.74012 | 732 | 2 | SH |
|  | Blue Ridge Pkwy | A71 | 37.14706 | -80.09796 | 874 | 3 | OMH |
|  | Cumberland Gap NHP | A78 | 36.65478 | -83.49667 | 488 | 3 | SH |
|  |  | A82 | 36.60884 | -83.63612 | 446 | 3 | SH |
| Kentucky | Cumberland Gap NHP | A80 | 36.60814 | -83.67426 | 564 | 3 | MF |
| West Virginia | Monongahela NF | A104 | 39.12659 | -79.58440 | 1041 | 3 | SH |
|  |  | A105 | 39.11694 | -79.59679 | 1050 | 3 | SH |
|  |  | A102 | 38.88536 | -79.46547 | 980 | 3 | SH |
|  |  | A106 | 38.82374 | -79.38618 | 528 | 3 | OMH |
|  |  | A111 | 38.73560 | -79.60076 | 1045 | 3 | OMH |
|  |  | A112 | 38.72562 | -79.67355 | 991 | 3 | SH |
|  |  | A100 | 37.91928 | -80.27489 | 614 | 3 | SH |
