# Supplementary material for: Genetic insights into family group co-occurrence in Cryptocercus punctulatus, a sub-social woodroach from the southern Appalachian Mountains
Source: PeerJ. 2017 Mar 23;5:e3127. doi: 10.7717/peerj.3127 (PMC5366060; doi:10.7717/peerj.3127)
Supplement: Appendix S2 [file peerj-05-3127-s002.docx]

**Garrick RC (2017) Genetic insights into family group co-occurrence in *Cryptocercus punctulatus*, a sub-social woodroach from the southern Appalachian Mountains. *PeerJ*.**

**Appendix 2**. Individual-based GenBank accession numbers for each *C. punctulatus* woodroach included in this study. The sex of each individual is abbreviated as M (male) or F (female), and the name of each sequenced mitochondrial DNA region is abbreviated as *COI* (cytochrome oxidase subunit I) or *COII* (cytochrome oxidase subunit II).

| **Individual ID** | **Sex** | **COI** | **COII** | **Individual ID** | **Sex** | **COI** | **COII** |
| --- | --- | --- | --- | --- | --- | --- | --- |
| CpA05_2 | M | KX944872 | KX945115 | CpA68_1 | F | KX945043 | KX945286 |
| CpA05_3 | F | KX944873 | KX945116 | CpA68_2 | F | KX945044 | KX945287 |
| CpA06_1 | F | KX944874 | KX945117 | CpA68_3 | M | KX945045 | KX945288 |
| CpA06_2 | M | KX944875 | KX945118 | CpA69_1 | F | KX945046 | KX945289 |
| CpA06_3 | F | KX944876 | KX945119 | CpA69_2 | M | KX945047 | KX945290 |
| CpA08_1 | ? | KX944877 | KX945120 | CpA71_1 | F | KX945048 | KX945291 |
| CpA08_2 | F | KX944878 | KX945121 | CpA71_2 | F | KX945049 | KX945292 |
| CpA09_1 | F | KX944879 | KX945122 | CpA71_3 | M | KX945050 | KX945293 |
| CpA09_2 | F | KX944880 | KX945123 | CpA75_1 | F | KX944901 | KX945144 |
| CpA09_3 | F | KX944881 | KX945124 | CpA75_2 | M | KX944902 | KX945145 |
| CpA10_1 | F | KX944882 | KX945125 | CpA78c_1 | F | KX945097 | KX945340 |
| CpA10_2 | F | KX944883 | KX945126 | CpA78c_2 | F | KX945098 | KX945341 |
| CpA11_1 | F | KX944884 | KX945127 | CpA78c_3 | F | KX945099 | KX945342 |
| CpA11_2 | F | KX944885 | KX945128 | CpA80_1 | F | KX944903 | KX945146 |
| CpA11_3 | M | KX944886 | KX945129 | CpA80_2 | M | KX944904 | KX945147 |
| CpA16_1 | F | KX944887 | KX945130 | CpA80_3 | F | KX944905 | KX945148 |
| CpA16_2 | F | KX944888 | KX945131 | CpA82_1 | F | KX945100 | KX945343 |
| CpA16_3 | F | KX944889 | KX945132 | CpA82_2 | F | KX945101 | KX945344 |
| CpA17_1 | F | KX944970 | KX945213 | CpA82_3 | M | KX945102 | KX945345 |
| CpA17_2 | M | KX944971 | KX945214 | CpA85_1 | F | KX944906 | KX945149 |
| CpA17_3 | F | KX944972 | KX945215 | CpA85_2 | M | KX944907 | KX945150 |
| CpA18_1 | F | KX944973 | KX945216 | CpA85_3 | F | KX944908 | KX945151 |
| CpA18_2 | M | KX944974 | KX945217 | CpA90_1 | M | KX944909 | KX945152 |
| CpA18_3 | F | KX944975 | KX945218 | CpA90_2 | M | KX944910 | KX945153 |
| CpA19_1 | F | KX944976 | KX945219 | CpA90_3 | F | KX944911 | KX945154 |
| CpA19_2 | F | KX944977 | KX945220 | CpA92_1 | F | KX944912 | KX945155 |
| CpA19_3 | F | KX944978 | KX945221 | CpA92_2 | M | KX944913 | KX945156 |
| CpA20_1 | F | KX944979 | KX945222 | CpA93_1 | M | KX945007 | KX945250 |
| CpA20_2 | F | KX944980 | KX945223 | CpA93_2 | M | KX945008 | KX945251 |
| CpA20_3 | F | KX944981 | KX945224 | CpA93_3 | F | KX945009 | KX945252 |
| CpA21_1 | F | KX944982 | KX945225 | CpA94_1 | M | KX945103 | KX945346 |
| CpA21_2 | M | KX944983 | KX945226 | CpA94_2 | M | KX945104 | KX945347 |
| CpA22_1 | M | KX944984 | KX945227 | CpA94_3 | M | KX945105 | KX945348 |
| CpA22_2 | F | KX944985 | KX945228 | CpA95_1 | F | KX944914 | KX945157 |
| CpA22_3 | M | KX944986 | KX945229 | CpA95_2 | M | KX944915 | KX945158 |
| CpA23_1 | F | KX944987 | KX945230 | CpA95_3 | F | KX944916 | KX945159 |
| CpA23_2 | M | KX944988 | KX945231 | CpA96_1 | M | KX945106 | KX945349 |
| CpA23_3 | F | KX944989 | KX945232 | CpA96_2 | F | KX945107 | KX945350 |
| CpA25_1 | M | KX944990 | KX945233 | CpA96_3 | F | KX945108 | KX945351 |
| CpA25_2 | F | KX944991 | KX945234 | CpA97_1 | M | KX945109 | KX945352 |
| CpA25_3 | F | KX944992 | KX945235 | CpA97_2 | M | KX945110 | KX945353 |
| CpA27_1 | M | KX944993 | KX945236 | CpA97_3 | F | KX945111 | KX945354 |
| CpA27_2 | F | KX944994 | KX945237 | CpA98_1 | F | KX945112 | KX945355 |
| CpA27_3 | M | KX944995 | KX945238 | CpA98_2 | M | KX945113 | KX945356 |
| CpA28_1 | M | KX945071 | KX945314 | CpA98_3 | F | KX945114 | KX945357 |
| CpA28_2 | F | KX945072 | KX945315 | CpA100_1 | F | KX945051 | KX945294 |
| CpA28_3 | F | KX945073 | KX945316 | CpA100_2 | M | KX945052 | KX945295 |
| CpA31_1 | F | KX944890 | KX945133 | CpA100_3 | F | KX945053 | KX945296 |
| CpA31_2 | M | KX944891 | KX945134 | CpA102_1 | F | KX945054 | KX945297 |
| CpA31_3 | F | KX944892 | KX945135 | CpA102_2 | M | KX945055 | KX945298 |
| CpA32_1 | F | KX944893 | KX945136 | CpA102_3 | M | KX945056 | KX945299 |
| CpA32_2 | F | KX944894 | KX945137 | CpA104_1 | F | KX945057 | KX945300 |
| CpA32_3 | F | KX944895 | KX945138 | CpA104_2 | F | KX945058 | KX945301 |
| CpA33_1 | F | KX944996 | KX945239 | CpA104_3 | F | KX945059 | KX945302 |
| CpA33_2 | M | KX944997 | KX945240 | CpA105b_1 | M | KX945060 | KX945303 |
| CpA34_1 | F | KX944998 | KX945241 | CpA105b_2 | M | KX945061 | KX945304 |
| CpA34_2 | M | KX944999 | KX945242 | CpA106_1 | F | KX945062 | KX945305 |
| CpA34_3 | F | KX945000 | KX945243 | CpA106_2 | M | KX945063 | KX945306 |
| CpA35_1 | F | KX945001 | KX945244 | CpA106_3 | M | KX945064 | KX945307 |
| CpA35_2 | M | KX945002 | KX945245 | CpA111_1 | F | KX945065 | KX945308 |
| CpA35_3 | M | KX945003 | KX945246 | CpA111_2 | M | KX945066 | KX945309 |
| CpA36_1 | F | KX945074 | KX945317 | CpA111_3 | F | KX945067 | KX945310 |
| CpA36_2 | M | KX945075 | KX945318 | CpA112_1 | F | KX945068 | KX945311 |
| CpA36_3 | M | KX945076 | KX945319 | CpA112_2 | F | KX945069 | KX945312 |
| CpA37b_1 | F | KX945077 | KX945320 | CpA112_3 | M | KX945070 | KX945313 |
| CpA37b_2 | M | KX945078 | KX945321 | CpA116_1 | M | KX944917 | KX945160 |
| CpA38_1 | M | KX944896 | KX945139 | CpA116_2 | F | KX944918 | KX945161 |
| CpA38_2 | M | KX944897 | KX945140 | CpA116_3 | F | KX944919 | KX945162 |
| CpA40_1 | F | KX945004 | KX945247 | CpA117_1 | M | KX944920 | KX945163 |
| CpA40_2 | F | KX945005 | KX945248 | CpA117_2 | M | KX944921 | KX945164 |
| CpA40_3 | M | KX945006 | KX945249 | CpA117_3 | M | KX944922 | KX945165 |
| CpA41_1 | M | KX944898 | KX945141 | CpA118b_1 | F | KX944923 | KX945166 |
| CpA41_2 | M | KX944899 | KX945142 | CpA118b_2 | M | KX944924 | KX945167 |
| CpA41_3 | F | KX944900 | KX945143 | CpA118b_3 | M | KX944925 | KX945168 |
| CpA44_1 | F | KX945079 | KX945322 | CpA119_1 | F | KX944926 | KX945169 |
| CpA44_2 | F | KX945080 | KX945323 | CpA119_2 | M | KX944927 | KX945170 |
| CpA46_1 | M | KX945081 | KX945324 | CpA119_3 | M | KX944928 | KX945171 |
| CpA46_2 | M | KX945082 | KX945325 | CpA120_1 | F | KX944929 | KX945172 |
| CpA46_3 | F | KX945083 | KX945326 | CpA120_2 | M | KX944930 | KX945173 |
| CpA47_1 | M | KX945084 | KX945327 | CpA120_3 | F | KX944931 | KX945174 |
| CpA47_2 | M | KX945085 | KX945328 | CpA122b_1 | F | KX944932 | KX945175 |
| CpA47_3 | M | KX945086 | KX945329 | CpA122b_2 | M | KX944933 | KX945176 |
| CpA48_1 | M | KX945087 | KX945330 | CpA122b_3 | M | KX944934 | KX945177 |
| CpA48_2 | M | KX945088 | KX945331 | CpA127_1 | F | KX944935 | KX945178 |
| CpA48_3 | M | KX945089 | KX945332 | CpA127_2 | M | KX944936 | KX945179 |
| CpA49_1 | F | KX945090 | KX945333 | CpA128_1 | M | KX944937 | KX945180 |
| CpA49_2 | F | KX945091 | KX945334 | CpA128_2 | F | KX944938 | KX945181 |
| CpA50_1 | M | KX945092 | KX945335 | CpA128_3 | F | KX944939 | KX945182 |
| CpA50_2 | M | KX945093 | KX945336 | CpA129_1 | M | KX944940 | KX945183 |
| CpA51_1 | F | KX945094 | KX945337 | CpA129_2 | F | KX944941 | KX945184 |
| CpA51_2 | F | KX945095 | KX945338 | CpA129_3 | M | KX944942 | KX945185 |
| CpA51_3 | M | KX945096 | KX945339 | CpA131_1 | M | KX944943 | KX945186 |
| CpA54_1 | F | KX945013 | KX945256 | CpA131_2 | F | KX944944 | KX945187 |
| CpA54_2 | F | KX945014 | KX945257 | CpA131_3 | M | KX944945 | KX945188 |
| CpA54_3 | F | KX945015 | KX945258 | CpA132b_1 | F | KX944946 | KX945189 |
| CpA57_1 | F | KX945016 | KX945259 | CpA132b_2 | F | KX944947 | KX945190 |
| CpA57_2 | M | KX945017 | KX945260 | CpA133b_1 | M | KX944948 | KX945191 |
| CpA57_3 | F | KX945018 | KX945261 | CpA133b_2 | F | KX944949 | KX945192 |
| CpA58_1 | M | KX945019 | KX945262 | CpA134b_1 | F | KX944950 | KX945193 |
| CpA58_2 | M | KX945020 | KX945263 | CpA134b_2 | F | KX944951 | KX945194 |
| CpA58_3 | F | KX945021 | KX945264 | CpA134b_3 | M | KX944952 | KX945195 |
| CpA59_1 | F | KX945022 | KX945265 | CpA135_1 | F | KX944953 | KX945196 |
| CpA59_2 | M | KX945023 | KX945266 | CpA135_2 | F | KX944954 | KX945197 |
| CpA59_3 | F | KX945024 | KX945267 | CpA135_3 | M | KX944955 | KX945198 |
| CpA61_1 | F | KX945025 | KX945268 | CpA136_1 | F | KX944956 | KX945199 |
| CpA61_2 | M | KX945026 | KX945269 | CpA136_2 | M | KX944957 | KX945200 |
| CpA61_3 | F | KX945027 | KX945270 | CpA137b_1 | M | KX944958 | KX945201 |
| CpA62_1 | F | KX945028 | KX945271 | CpA137b_2 | F | KX944959 | KX945202 |
| CpA62_2 | M | KX945029 | KX945272 | CpA137b_3 | F | KX944960 | KX945203 |
| CpA62_3 | M | KX945030 | KX945273 | CpA138b_1 | F | KX944961 | KX945204 |
| CpA63_1 | M | KX945031 | KX945274 | CpA138b_2 | M | KX944962 | KX945205 |
| CpA63_2 | F | KX945032 | KX945275 | CpA138b_3 | F | KX944963 | KX945206 |
| CpA63_3 | F | KX945033 | KX945276 | CpA142c_1 | F | KX944964 | KX945207 |
| CpA65_1 | M | KX945034 | KX945277 | CpA142c_2 | M | KX944965 | KX945208 |
| CpA65_2 | M | KX945035 | KX945278 | CpA142c_3 | M | KX944966 | KX945209 |
| CpA65_3 | F | KX945036 | KX945279 | CpA143_1 | F | KX944967 | KX945210 |
| CpA66_1 | F | KX945037 | KX945280 | CpA143_2 | F | KX944968 | KX945211 |
| CpA66_2 | M | KX945038 | KX945281 | CpA143_3 | F | KX944969 | KX945212 |
| CpA66_3 | M | KX945039 | KX945282 | CpA146c_1 | F | KX945010 | KX945253 |
| CpA67_1 | F | KX945040 | KX945283 | CpA146c_2 | M | KX945011 | KX945254 |
| CpA67_2 | F | KX945041 | KX945284 | CpA146c_3 | F | KX945012 | KX945255 |
| CpA67_3 | M | KX945042 | KX945285 |  |  |  |  |
